# Ultrahigh Water Permeance of Reduced Graphene Oxide Membrane for Radioactive Liquid Waste Treatment

**DOI:** 10.3390/membranes11110809

**Published:** 2021-10-24

**Authors:** Xinming Xia, Feng Zhou, Risheng Yu, Longsheng Cao, Liang Chen

**Affiliations:** 1 Department of Optical Engineering, Zhejiang Province Key Laboratory Carbon Cycling Forest Ecosy, Zhejiang A&F University, Hangzhou 311300, China; xinming_xia@163.com (X.X.); 18279440234@163.com (R.Y.); 2 Radiation Monitoring Technical Center of Ministry of Ecology and Environment, Key Laboratory of Radiation Environmental Safety Monitoring of Zhejiang Province, State Environmental Protection Key Laboratory of Radiation Environmental Monitoring, Hangzhou 310012, China; c13567193570@163.com; 3 School of Physical Science and Technology, Ningbo University, Ningbo 315211, China

**Keywords:** reduced graphene oxide, membrane, radioactive liquid waste, nanofiltration, permeance

## Abstract

Membrane methods exhibit great potential for application in radioactive liquid waste treatment. In this work, we prepared a reduced graphene oxide using the amino-hydrothermal method (AH-rGO) that exhibited effective rejection rates of 99.9% for CoCl_2_, ZnCl_2_, NiCl_2_, and radionuclide ^60^Co solutions with an ultrahigh water permeance of >71.9 L m^−2^ h^−1^ bar^−1^. The thickness of the AH-rGO membranes affects the water permeance, as the membrane with a thickness of ≈250 nm has the highest water permeance of up to 125.1 L m^−2^ h^−1^ bar^−1^ with the corresponding rejection rate of 86.8%. Importantly, this is the most permeable membrane with a satisfactory level of the rejection rate for typical radioactive ions of Co^2+^, Zn^2+^, and Ni^2+^. Moreover, the AH-rGO membranes presented excellent stability. These findings demonstrate the potential of reduced graphene oxide (rGO) membranes for radioactive liquid waste treatment.

## 1. Introduction

Nuclear power plants have seen steady growth in recent years as an essential element of a reliable and low-carbon electricity supply [1]. With the continuous development of nuclear energy, a large amount of nuclear waste has been produced, which has potentially harmful effects on human beings. Radioactive nuclides, such as ^90^Sr, ^137^Cs, ^60^Co, ^65^Zn, and ^63^Ni, are primary species generated in the process of nuclear power production and let out in an unexpected accident such as the explosion at the Fukushima Daiichi power plant in 2011 [2,3,4]. Due to its harmfulness, the radioactive liquid waste must be pre-sorted and treated to reduce the volume of radioactive liquid waste to the smallest possible volume for long-term storage or final disposal [5]. Various technologies have been applied for the treatment of radioactive liquid waste, such as precipitation, adsorption, membrane separation, and other methods [6,7,8,9,10,11]. Membrane methods for separating liquid mixtures have been intensively developed with promising results to resolve various scientific and environmental problems [12,13,14,15,16,17]. However, excellent ions rejection of membranes leads to a reduction in water permeability and vice versa [18], since smaller pore sizes always mean higher selectivity but limited water permeability [19,20].

Reduced graphene oxide (rGO) membranes have promising application as nanofiltration (NF) membranes due to their superior filtration performance in water permeance and stability [21,22,23,24,25,26,27]. There are interlayer spacings of the GO membranes stacked by graphene oxide sheets, which have well-defined nanometer pores and can exhibit low frictional water channels [17]. Moreover, the variable interlayer spacings of GO membranes can be controlled by chemical cross-linking [28,29] or physical encapsulating [30], and they are especially controlled by cationic control of the interlayer spacing with ångström precision [16], resulting in high rejection rates for ions with small size. For typical radionuclides in radioactive liquid waste, the filtration performance of rGO membranes has great potential to be greatly desirable but needs systematic research to be addressed. For example, the sharp decrease in the oxygen functional groups in GO membranes after various reduction may lead to a low rejection rate [25].

Here, we achieved an ultrahigh water permeance while still maintaining a high rejection rate for typical radioactive liquid waste using amino-hydrothermal rGO (AH-rGO) membranes. The effective rejection rates reached up to 99.9% for Co^2+^, Zn^2+^, Ni^2+^, and radionuclide ^60^Co solutions with an ultrahigh water permeance of >71.9 L m^−2^ h^−1^ bar^−1^, which is superior filtration performance compared to other membranes, as the maximum water permeability of the membrane was about 25.0 L m^−2^ h^−1^ bar^−1^ with a rejection rate of 95.7% for Zn^2+^ ions [31]. Furthermore, the AH-rGO membranes showed outstanding rejection stability in different ions concentration, membrane thickness, and long-term operation, using non-radioactive CoCl_2_ solutions as an example. Meanwhile, the water permeance increased with decreasing membrane thickness, and the AH-rGO membrane with a thickness of ≈250 nm had the highest water permeance of up to 125.1 L m^−2^ h^−1^ bar^−1^ with the corresponding rejection rate of 86.8%. The results show that AH-rGO membranes have potential in applications of radioactive liquid waste treatment.

## 2. Materials and Methods

### 2.1. Materials

Cobalt (II) chloride hexahydrate (CoCl_2_ 6H_2_O), zinc chloride (ZnCl_2_), and nickel (II) chloride hexahydrate (NiCl_2_ 6H_2_O) were purchased from Sinopharm Chemical Reagent Co. (Shanghai, China). Radionuclide ^60^Co standard solution was purchased from the National Institute of Metrology (Beijing, China). Deionized (DI) water (18.2 MΩ) was utilized in all the experiments. All the reagents were utilized as received without further purification.

### 2.2. Preparation of AH-rGO Suspension

The AH-rGO suspensions were prepared by the amino-hydrothermal method [27,32,33]. Graphite powders were concentrated in H_2_SO_4_ containing K_2_S_2_O_8_ and P_2_O_5_ and stirred continuously for several hours. The mixture was washed and filtered in DI water. After vacuum drying, pre-oxidized graphite was obtained. Pre-oxidized graphite was oxidized in concentrated H_2_SO_4_ and KMnO_4_ at 60 °C and diluted with DI water at 80 °C. Then, the product was further oxidized with H_2_O_2_ and washed using HCl aqueous solution and DI water. The GO suspension was obtained for further use: 52.5 mL of 2 mg/mL GO suspension was mixed with 490 mL DI water and 360 mL 28% NH_4_OH. The mixed solution was stirred at 80 °C for 6 h and further stirred at 90 °C for 1 h. Finally, the AH-rGO suspension was obtained for further use.

### 2.3. Filtration Experiments

The AH-rGO membranes were prepared by vacuum filtration of 40 mL of 33.0 mg/L AH-rGO suspension on the mixed cellulose ester (MCE; 0.22 μm, JINTENG, Hangzhou, China) substrate. The GO membranes (40 mL of 33.0 mg/L GO suspension) were prepared according to the above steps. First, 100 mL 50 mg/L CoCl_2_, ZnCl_2_, or NiCl_2_ solution was added to the feed side of the terminal filter unit, respectively. Then, the solutions were filtered through AH-rGO membrane at a pressure of 1 bar. The filtrates were obtained 10 min after the filtration was stable.

The ^60^Co was selected for the radioactive filtration experiment. Similar to the filtration experiments, the 100 mL mixtures of 50 mg/L CoCl_2_ and 399 Bq/L ^60^Co were added to the feed side.

Finally, water permeance (*J_W_*, L m^−2^ h^−1^ bar^−1^) and rejection rates (*R*, %) were calculated according to Equations (1) and (2).

(1) $$J_{W}=\frac{V}{\Delta t\times A\times P}$$

(2) $$R=\left(\frac{1-C_{P}}{C_{f}}\right)\times 100\%$$

where *V* is the volume of the filter liquor (L), ∆*t* is the permeance time (h), *A* is the effective membrane area (*A* = 1.134 × 10^−3^ m^2^), and *P* is the filtration pressure (*P* = 1 bar). *C_p_* and *C_f_* are the concentrations of filtrate and feed ions solutions, respectively, which were measured by inductive coupled plasma-optical emission spectrometry (ICP-OES, iCAP 7400, Thermo Fisher Scientific, Dreieich, Germany). Alternatively, *C_p_* and *C_f_* are the concentrations of radioactive activity of the filtrate and the feed solution, respectively, which were determined by high-purity germanium γ spectrometer (GMX–60, ORTEC, Atlanta, GA, USA).

### 2.4. Characterizations

The morphology of the membrane was analyzed by Scanning Electron Microscope (SEM). The chemical compositions were analyzed by X-ray photoelectron spectroscopy (XPS). The chemical functional groups were analyzed by Fourier transform infrared (FT-IR) spectra. The absorbance spectra of AH-rGO suspension were analyzed by ultraviolet-visible spectroscopy (UV-Vis). The static contact angle was measured by a Telescopic goniometer. The surface charge of the AH-rGO suspensions was measured by Zeta potential. The details of instrumental analysis can be found in the Supplementary Materials Section 1.

## 3. Results and Discussion

### 3.1. Characterizations of the AH-rGO

**Figure 1:**
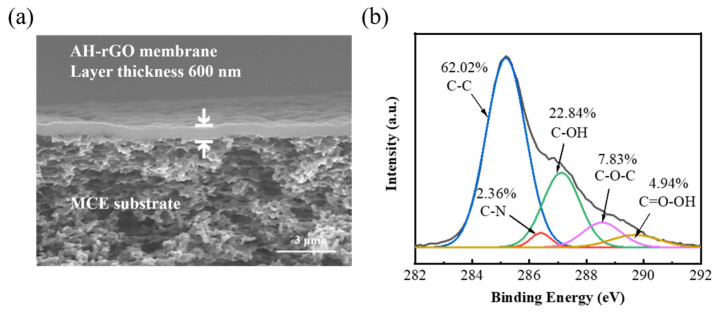
(**a**) SEM image of AH-rGO membrane. (**b**) XPS spectra of C1s for AH-rGO membrane.

The zeta potential of the AH-rGO suspensions was –34.1 ± 3.2 mV, showing a good dispersion stability of nanosheets. The morphologies of the GO and AH-rGO membranes were characterized by SEM image (Figure S1a,b). As shown in Figure 1a, the thickness of the membrane prepared with 40 mL of 33.0 mg/L AH-rGO suspensions was ≈600 nm in the dry state. It clearly shows a defect-free surface and uniform thickness, which helps to improve water flux and the ions rejection rate [25,33,34]. The average static contact angle of the AH-rGO membrane surface was 57.8° (see Figure S2a), indicating that the membrane has good hydrophilicity and thus contributes to water permeance [35].

The chemical compositions of the GO and AH-rGO membranes were analyzed by XPS spectra of C1s. As shown in Figure 1b, the C1s of the XPS spectrum of AH-rGO is divided into five Gaussian peaks at 285.18, 286.4, 287.12, 288.54, and 289.7 eV, corresponding to the typical signals of C=C, C-N, C-OH, C-O-C, and O=C-OH, respectively. The C-N compositions (2.36%) and wide scan of XPS spectra of GO and AH-rGO (see Figure S1c) indicated the presence of C-N on the reduced GO surface by an ammonia reduction process. Further, the FT-IR spectra and UV-vis absorption spectra were analyzed to verify the structures of the AH-rGO membranes. As shown in Figure S2b, the C-N stretching vibrations were observed at ≈1232 cm^−1^, which is consistent with our XPS results. The UV-vis absorption spectra (see Figure S2c) showed that the characteristic peaks at ≈230 nm were attributed to π–π* from the aromatic rings. The results are consistent with the previous reports [27,36,37,38].

### 3.2. Membrane Rejection of Typical Radioactive Ions

**Figure 2:**
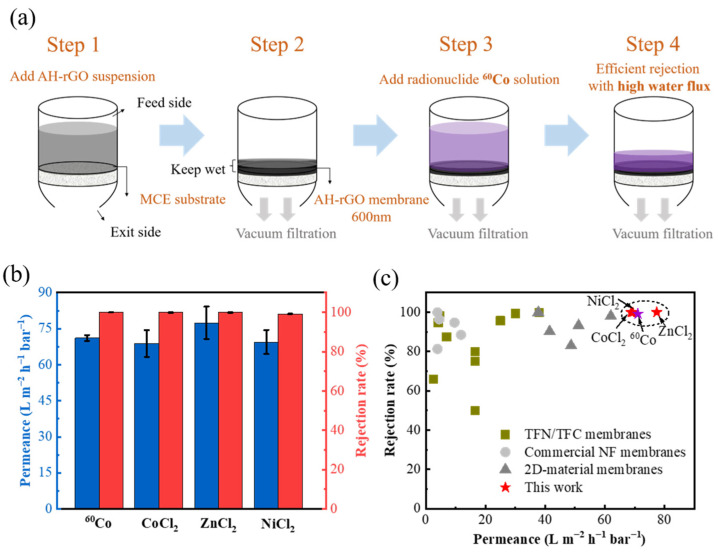
(**a**) A schematic of the filtration process. (**b**) Water permeance and rejection rate of AH-rGO membranes for 399 Bq/L radionuclide ^60^Co and 50 mg/L CoCl_2_, ZnCl_2_, NiCl_2_ solutions. (**c**) Comparisons of different nanofiltration membranes in water permeance and rejection rates for the ions (Co^2+^, Zn^2+^, Ni^2+^, and the typical divalent ion) in the literature.

Filtration experiments of typical radioactive ions by the AH-rGO membranes were performed. In order to improve water performance, the membranes were treated without drying [39]. The detailed process of the filtration is shown in Figure 2a. According to Step 1, the AH-rGO membrane was obtained on MCE through vacuum filtration. Then, we kept the membrane surface wet and added the radionuclide ^60^Co solution (Step 2 and 3), and finally collected the filtrate at the exit side through vacuum filtration (Step 4).

As shown in Figure 2b, for 50 mg/L CoCl_2_ and 399 Bq/L ^60^Co mixed solutions, the water permeance was 71.1 L m^−2^ h^−1^ bar^−1^ with a high rejection rate of 99.2% through AH-rGO membranes under a pressure of 1 bar. When the 50 mg/L CoCl_2_ solution is replaced by radionuclide ^60^Co solution in equal amount, its specific radioactivity is about 9.6 × 10^11^ Bq/L. Then, filtration experiments were further performed for other non-radioactive ions rejected by the AH-rGO membranes. The rejection rates for 50 mg/L of CoCl_2_, ZnCl_2_, or NiCl_2_ solutions were 99.9%, 99.9%, and 99.9%, while the water permeance was 68.8, 77.4, and 69.4 L m^−2^ h^−1^ bar^−1^, respectively, showing high water permeance and rejection. We attribute the excellent performance to the partial reduction of GO sheets, resulting in the AH-rGO membrane having two types of regions: functionalized (oxidized) and pristine [17]. The oxidized regions act as spacers that help water to intercalate the interlayer spacings, whereas pristine regions allow nearly frictionless flow of water [17].

We noted that the radioactive wastewater, which contains various radionuclides (such as ^60^Co, ^65^Zn, ^63^Ni, etc.), have valences and hydration sizes similar to those of non-radioactive ions. The water permeance and rejections of ions (Co^2+^, Zn^2+^, and Ni^2+^) in the AH-rGO membrane reported in this paper were further compared, as shown in Figure 2c and Table S1. Our results presented superior filtration performance compared to other membranes, as the maximum water permeability of the membrane was about 25.0 L m^−2^ h^−1^ bar^−1^ with a rejection rate of 95.7% for Zn^2+^ ions [31]. We further performed rejection experiments on the pure GO membrane as a control for 50 mg/L CoCl_2_ solution. As shown in Figure S3, for pure GO membranes, the water permeance was only 17.1 L m^−2^ h^−1^ bar^−1^ with a low rejection rate of 36.7% under a pressure of 1 bar. Meanwhile, the water permeance of the AH-rGO membrane was 68.8 L m^−2^ h^−1^ bar^−1^ with a high rejection rate of 99.9%, showing that our AH-rGO membrane has superior filtration performance for typical radioactive ions not only for other NF membranes (Figure 2c) but also GO membranes. Thus, the filtration performances of the AH-rGO membranes in this paper were superior to those of the most advanced separation membranes for radionuclide ^60^Co and ions (Co^2+^, Zn^2+^, and Ni^2+^) rejection. Therefore, the AH-rGO membranes have potential application in the removal of radioactive wastewater.

### 3.3. Performance of AH-rGO Membranes

**Figure 3:**
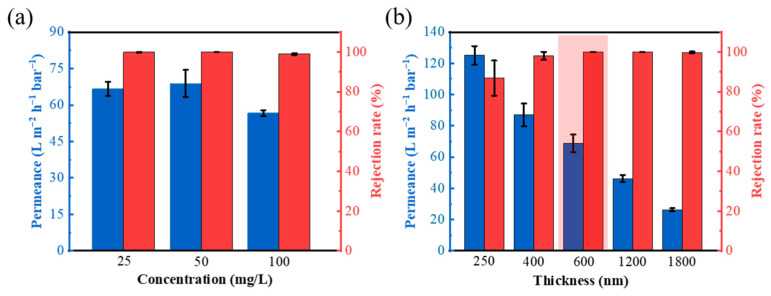
(**a**) Water permeance and rejection of CoCl_2_ solutions through the AH-rGO membranes as a function of ion concentration. (**b**) Water permeance and rejection of 50 mg/L CoCl_2_ solutions through the AH-rGO membranes as a function of thickness. The selected optimal thickness is highlighted by the region shaded red.

In order to further systemically evaluate the membrane performance, we further analyze the effect of solution concentration, membrane thickness, and filtration time on the filtration performance of AH-rGO membranes, using non-radioactive CoCl_2_ solutions as an example. The water permeance and rejection of CoCl_2_ solutions with different concentrations (25 to 100 mg/L) showed outstanding stability, as shown in Figure 3a. There is a slight decrease in water permeance with the increase in solution concentration, which we attribute to the potential for the increased salt concentration to affect the membrane performance due to concentration polarization [40,41]. However, the water permeance of 54.6 L m^−2^ h^−1^ bar^−1^ with a rejection rate of 99.0% was still superior to that of the most advanced separation membranes. The performances of AH-rGO membranes with different reduction degrees were also measured (details in Supplementary Materials Section 5), as shown in Figure S4, indicating that the current preparation method of AH-rGO in this paper is suitable. In addition, the effect of ion adsorption on the AH-rGO membrane for Co^2+^ indicated that the significant effect on the removal of Co^2+^ ions is mainly due to rejection by the AH-rGO membranes (in Supplementary Materials Section 6).

AH-rGO membranes with different thicknesses were prepared by controlling the concentration of AH-rGO suspension. According to the membranes preparation method of the above experiments (membranes thickness 600 nm, Figure 1a), membranes of different thickness (250–1800 nm) were prepared (see Supplementary Materials Section 7). As shown in Figure 3b, the water permeance decreases gradually with the increase in membrane thickness. When the membrane thickness was 250 nm, the water permeance reached up to 125.1 L m^−2^ h^−1^ bar^−1^, with a lower rejection rate (86.8%) compared with other membrane thickness. Taking into account the demands of the efficient removal of radionuclides, the AH-rGO membrane of 600 nm should be the most suitable design (the region shaded red in Figure 3b).

**Figure 4:**
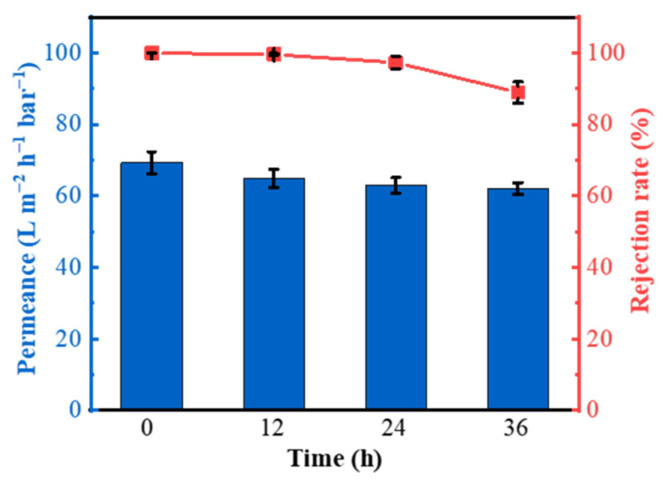
Effect of AH-rGO membranes on water permeance and rejection rates of 50 mg/L CoCl_2_ solution with different filtration times.

In addition, in order to investigate the stability of the AH-rGO membrane with a thickness of 600 nm in the permeation process, we took 50 mg/L CoCl_2_ solution as an example and measured the water permeance and rejection rate every 12 h employing a continuous process for 36 h under vacuum filtration. As shown in Figure 4, the AH-rGO membrane had a very stable rejection rate and water permeance, which decrease slightly after 12 h. The water permeance was stable at 62.0–69.3 L m^−2^ h^−1^ bar^−1^ for 36 h, and the rejection rate was always above ≈89.0%. The slightly decreasing at ≈89.0% was due to the concentration polarization caused by surface accumulation after a long period of interception in our dead-end filtration set up. Furthermore, the AH-rGO membranes demonstrated excellent aqueous stability, as they remained intact under ultrasound treatment for 120 min, as shown in Figure S5, indicating that the membrane can resist the swelling of GO membranes in water. From the excellent aqueous stability of the AH-rGO membranes under ultrasound treatment discussed above, a robust membrane with good antifouling property can be expected. We confirmed the good antifouling property using a semi-continuous antifouling experiment [39,42] (details in Supplementary Materials Section 9), as shown in Figure S6, which showed outstanding antifouling performance of the AH-rGO membranes. Therefore, the results presented the outstanding stability of the AH-rGO membrane.

## 4. Conclusions

In summary, AH-rGO membranes were prepared by the amino-hydrothermal method. The AH-rGO membranes presented ultrahigh water permeance and effective rejection rates for CoCl_2_, ZnCl_2_, NiCl_2_, and radionuclide ^60^Co solutions. Moreover, the AH-rGO membranes presented excellent stability. It is worthwhile to note that when the mass concentration is replaced by the activity concentration of the radionuclide in equal quantities, it is possible to achieve an efficient rejection of high-activity radioactive solutions with high water permeance. Overall, our work reveals the potential of rGO membranes in radioactive liquid waste treatment, owing to their effective radioactive ion rejection with ultrahigh permeance.

## Data Availability

The data presented in this study are available on request from the corresponding author.

## Supplementary Materials

The following are available online at https://www.mdpi.com/article/10.3390/membranes11110809/s1, Figure S1: SEM images ((a) and (b)) and XPS full-scan spectra (c) of GO and AH-rGO membranes. Figure S2: (a) Static contact angles of the AH-rGO membrane surface. (b) FT–IR spectra of GO and AH-rGO. (c) UV–vis absorption spectra of GO and AH-rGO. Figure S3: Water permeances and rejection rates of the GO and AH-rGO membranes for 50 mg/L CoCl_2_ solutions. All tests were repeated on three different samples, and the error was calculated from these three measurements. Figure S4: Filtration performance of AH-rGO membranes prepared with 70 °C, 80 °C, and 90 °C. Figure S5: Stability of AH-rGO and pure GO membrane in the aqueous solution treated with 40 kHz ultrasound for 120 min. Figure S6: Antifouling performance measurements of the AH-rGO membrane. The permeance and rejection rate of AH-rGO membrane for the 50 mg/L CoCl_2_ solution after surface and filtration cleaning with DI water. Table S1: Comparisons of different nanofiltration membranes in water permeance and rejection rates for the ions (Co^2+^, Zn^2+^, Ni^2+^, and the typical divalent ion) in the literature. References [43,44,45,46,47,48,49,50,51,52] have been cited in Supplementary Materials.
