# Supplementary material for: Ultrahigh Water Permeance of Reduced Graphene Oxide Membrane for Radioactive Liquid Waste Treatment
Source: Membranes (Basel). 2021 Oct 24;11(11):809. doi: 10.3390/membranes11110809 (PMC8624586; doi:10.3390/membranes11110809)
Supplement: Supplementary file 1 [file membranes-11-00809-s001.zip › membranes-1407682-supplementary.pdf]

membranes

MDPI

Supplementary Materials

Ultrahigh Water Permeance of Reduced Graphene Oxide Membrane for Radioactive Liquid Waste Treatment

Xinming Xia [1,†], Feng Zhou [2,*,†], Risheng Yu [1], Longsheng Cao [2] and Liang Chen [1,3,*]

[1] Department of Optical Engineering, Zhejiang Prov Key Lab Carbon Cycling Forest Ecosy, Zhejiang A&F University, Hangzhou 311300, China; xinming_xia@163.com (X.X.); 18279440234@163.com (R.Y.)
[2] Radiation Monitoring Technical Center of Ministry of Ecology and Environment, Key Laboratory of Radiation Environmental Safety Monitoring of Zhejiang Province, State Environmental Protection Key Laboratory of Radiation Environmental monitoring, Hangzhou 310012, China; c13567193570@163.com
[3] School of Physical Science and Technology, Ningbo University, Ningbo 315211, China
* Correspondence: zhoufeng150027@163.com (F.Z.); liangchen@zafu.edu.cn (L.C.)
† These authors contributed equally to this work.

Citation: Xia, X.; Zhou, F.; Yu, R.; Cao, L.; Chen, L. Ultrahigh Water Permeance of Reduced Graphene Oxide Membrane for Radioactive Liquid Waste Treatment. *Membranes* **2021**, *11*, 809. https://doi.org/10.3390/membranes11110809

Academic Editors: Xing Yang and Shuaifei Zhao

Received: 19 September 2021
Accepted: 21 October 2021
Published: 24 October 2021

Publisher's Note: MDPI stays neutral with regard to jurisdictional claims in published maps and institutional affiliations.

Copyright: © 2021 by the authors. Licensee MDPI, Basel, Switzerland. This article is an open access article distributed under the terms and conditions of the Creative Commons Attribution (CC BY) license (http://creativecommons.org/licenses/by/4.0/).

1. Instrumental analysis

The Scanning Electron Microscope (SEM) image of the membrane was taken on a Desktop Scanning Electron Microscope (SEM, TM4000, Japan). The chemical compositions of the membranes were analyzed by X-ray photoelectron spectroscopy (XPS, Thermo Scientific K-Alpha with Al Kα X-ray source at 1486.6 eV, America). The chemical functional groups of the membranes were analyzed by Fourier transformed infrared (FT-IR, Thermo Scientific Nicolet iS50, America) spectra in the range of 800–4000 cm^{-1}. The absorbance spectra of AH-rGO suspension (50mg/L) were measured by Ultraviolet-visible spectroscopy (UV-Vis, Shimadzu UV-Vis spectrophotometer UV2550, Japan). The static contact angle was measured by a Telescopic goniometer (JY-82B Kruss DSA, Germany) to analyze the membrane surface hydrophilicity. The surface charge of the AH-rGO suspensions (50 mg/L) was measured by Zeta potential (Brookhaven NanoBrook 90Plus PALS, America) to analyze the dispersion stabilities of nanosheets.

2. Characterizations

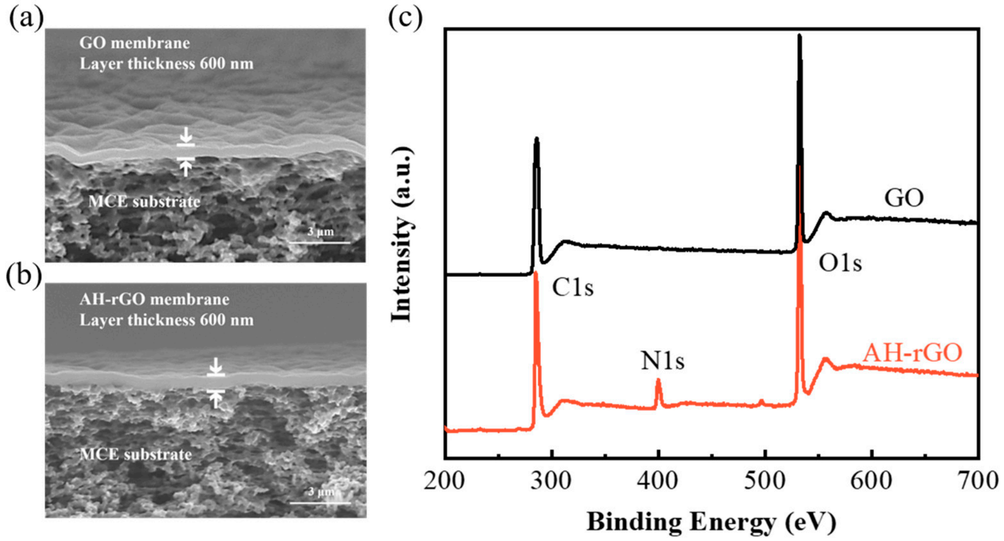

Figure S1. SEM images (**a** and **b**) and XPS full-scan spectra (**c**) of GO and AH-rGO membranes.

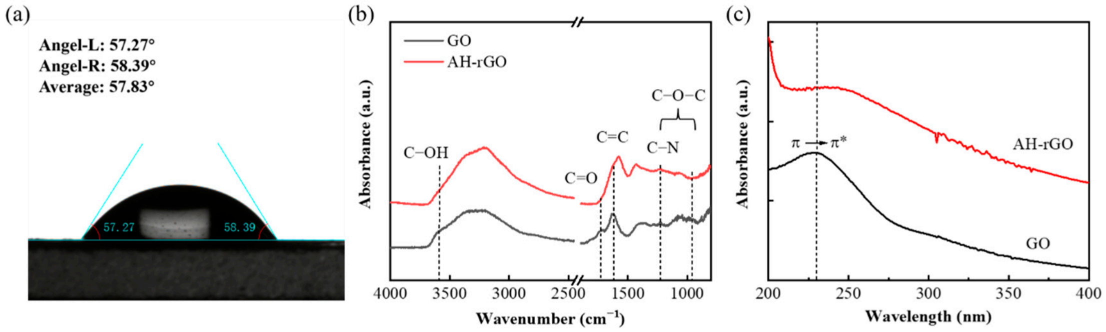

Figure S2. (**a**) Static contact angles of the AH-rGO membrane surface. (**b**) FT-IR spectra of GO and AH-rGO. (**c**) UV–vis absorption spectra of GO and AH-rGO.

3. Filtration performance of the nanofiltration membranes reported in literature in terms of water permeances and rejection rates

Table S1. Comparisons of different nanofiltration membranes in water permeance and rejection rates for the ions (Co^{2+}, Zn^{2+}, Ni^{2+}, and the typical divalent ion) in literature.

Membranes	Application	Water flux ($L\ m^{-2}\ h^{-1}\ bar^{-1}$)	Rejection Rate (%)	Type
GO/Torlon hollow fiber membrane [43]	Ni^{2+}, Zn^{2+}	4.7	95.9~98.1	TFC/TFN
HPEI-GO 10 [44]	Ni^{2+}	4.2	94.6	TFC/TFN
TFC /PES support [45]	$NiCl_2$	2.5	66.0	TFC/TFN
Polyamide nanofiltration membrane [46]	Co^{2+}	7	50~87.5	TFC/TFN
polyamide nanofiltration membrane [47]	Ni^{2+}	38	99.9	TFC/TFN
PES/B-Cur membranes [48]	Zn^{2+}, Ni^{2+}	30	99.11, 99.51	TFC/TFN
Polymer-grafted-carbon nanotube composite membranes [20]	$Co(NO_3)_2$, $Zn(NO_3)_2$, $Ni(NO_3)_2$	16.5	80, 75,50	TFC/TFN
positively charged composite hollow fiber nanofiltration membrane [31]	$ZnCl_2$	25.0	95.7	TFC/TFN
Filmtec-NF270 membrane [49]	Co^{2+}, Ni^{2+}	3.9	99.9, 81.3	Commercial membranes
NF90 membrane [50]	$Co(NO_3)_2$	4.5	96.4	Commercial membranes
NF270 membrane [50]	$Co(NO_3)_2$	11.8	88.5	Commercial membranes
ESNA1 membrane [50]	$Co(NO_3)_2$	9.6	94.7	Commercial membranes
GO/TiO2-PDDA [41]	$MgCl_2$	51.2	93.2	2D materials
TMV [51]	$MgSO_4$	62.0	98.0	2D materials
COF [52]	$MgCl_2$	41.5	90.2	2D materials
rGO membrane [27]	$ZnSO_4$	37.7	99.9	2D materials
GO membrane [39]	$ZnSO_4$	48.7	83.0	2D materials
**AH-rGO membrane**	**^{60}Co, $CoCl_2$, $ZnCl_2$, $NiCl_2$**	**125.1–68.8**	**86.8~99.9**	**This work**

4. Preparation of pure GO membranes and filtration

The pure GO membranes were prepared by vacuum filtration of 40 mL of 33.0 mg/L GO suspension on the mixed cellulose ester (MCE; 0.22 μm) substrate. 100 mL 50 mg/L $CoCl_2$ solution was added to the feed side, and filtered through pure GO membrane at a pressure of 1 bar. The filtrates were obtained 10 min after the filtration was stable.

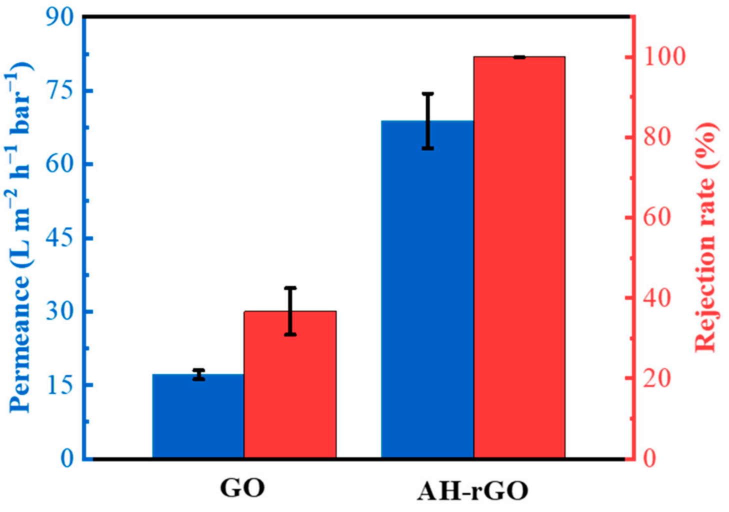

Figure S3. Water permeances and rejection rates of the GO and AH-rGO membranes for 50 mg/L $CoCl_2$ solutions. All tests were repeated on three different samples, and error was calculated from these three measurements.

As shown in Figure S3, for pure GO membranes, the water permeance was only 17.1 L m^{-2} h^{-1} bar^{-1} with a low rejection rate of 36.7 % under a pressure of 1 bar. While, the water permeance of AH-rGO membrane was 68.8 L m^{-2} h^{-1} bar^{-1} with a high rejection rate of 99.9%, showing that our AH-rGO membrane has superior filtration performance for typical radioactive ions not only for other NF membranes but also GO membranes.

5. Study of AH-GO membranes with different reduction degree

The performance of AH-rGO membranes with different reduction degree were also measured. In the preparation of AH-rGO suspension with different reduction degree, the GO suspensions were added to DI water and ammonium hydroxide, and the mixtures were stirred vigorously at 70 °C, 80 °C, and 90 °C, respectively. The membranes were further prepared by vacuum filtration on the MCE substrate. 100 mL 50 mg/L $CoCl_2$ solutions were added to the feed side for filtration experiments of the membranes with different reduction degree. As shown in Figure S4, the water permeance increased from 43.6 L m^{-2} h^{-1} bar^{-1} to 81.5 L m^{-2} h^{-1} bar^{-1} with the temperature of reduction. However, the rejection of membrane reduced at 90 °C decreased sharply, indicating that an excessive reduction at high temperature is not conducive to filtration performance of AH-rGO membranes.

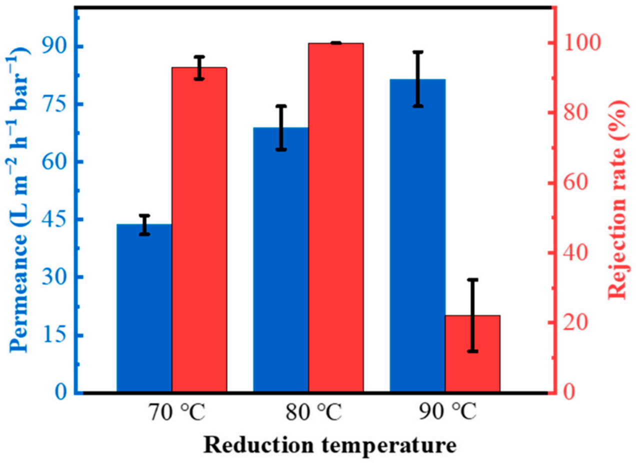

Figure S4. Filtration performance of AH-rGO membranes prepared with 70 °C, 80 °C, and 90 °C.

6. Effect of ion adsorption on AH-rGO membrane

We analysed the ion adsorption by the AH-rGO membranes with a thickness of ~600 nm in our filtration experiments. AH-rGO membrane was prepared on substrate using vacuum filtration. Then, 100 mL, 50 mg/L $CoCl_2$ solution was added to the feed side, respectively. These salt solutions under ambient conditions (without vacuum filtration) were stirred with a blender at ~180 RPM. Next, samples were collected after 30 min to evaluate the residual salt concentrations of the solutions.

The adsorption efficiencies of the AH-rGO membranes for $CoCl_2$ was only 3.1%. As mentioned in Figure 2b, the rejection rates of the AH-rGO membranes were 99.9%, which is much higher than 3.1% removed by adsorption. These results indicate that the significant effect on the removal of Co^{2+} ions is mainly due to rejection by the AH-rGO membranes.

7. Preparation of AH-GO membranes with different thickness

According to the membranes preparation method of the experiments (membranes thickness 600 nm), membranes of different thickness (250 nm–1800 nm) were prepared. AH-rGO membranes were prepared by 40 mL of 13.8 mg/L, 22 mg/L, 33 mg/L, 66 mg/L and 99 mg/L AH-rGO suspensions on MCE substrates using vacuum filtration, and corresponding a various thicknesses were 250, 400, 600, 1200 and 1800 nm, respectively.8. Ultrasound experiments to observe the stability of pure GO and AH-rGO membranes

Ultrasound experiments were performed to observe the stability of pure GO and AH-rGO membranes, as shown in Figure S5. GO membrane disintegrates within 1 min under ultrasound treatment, and is completely dispersed after 60 min. In contrast, the AH-rGO membranes remains intact under ultrasound treatment within 120 min. The AH-rGO membrane exhibited excellent aqueous stability, indicating that the membrane can well solve the swelling of GO membranes in water.

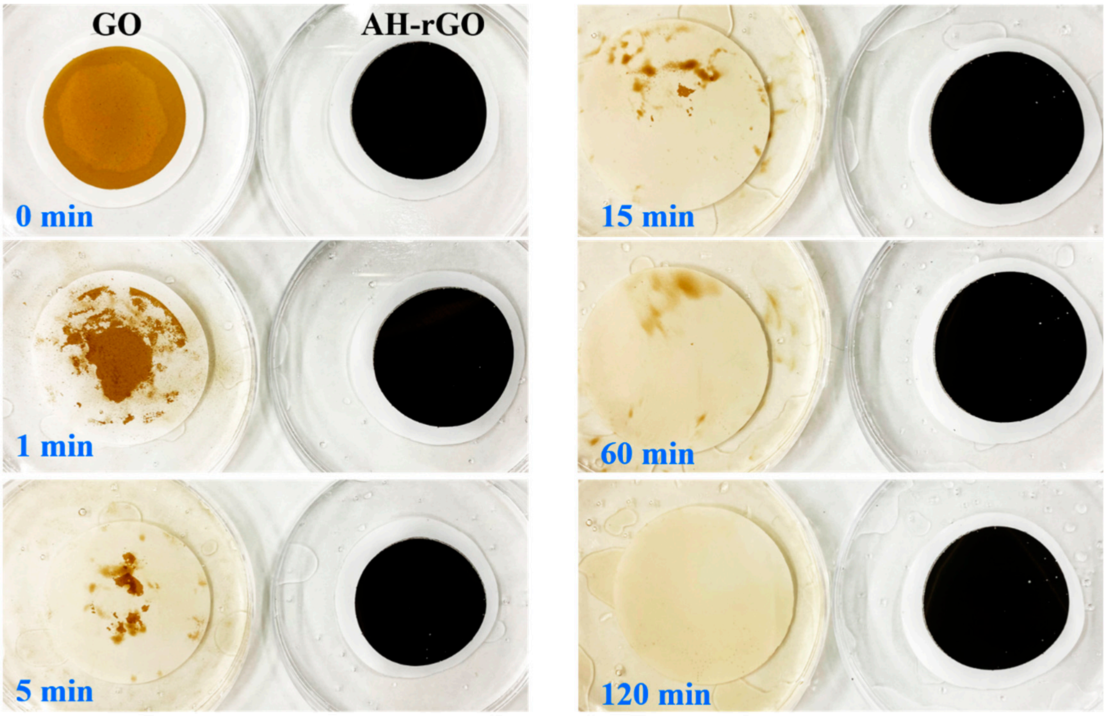

Figure S5. Stability of AH-rGO and pure GO membrane in the aqueous solution treated with 40 kHz ultrasound for 120 min.

9. Antifouling property of the AH-rGO membranes

Semi-continuous filtration experiments were performed to observe the antifouling property of the AH-rGO membranes. We noted that the feed salt concentration would continuously increase in the dead-end filtration set-up. The increased salt concentration (concentration polarization) could affect the membrane performance. Therefore, a semi-continuous process was employed for the antifouling experiment of the 50 mg/L $CoCl_2$ solution filtered by the AH-rGO membranes. Similar methods were used recently [39,42]. In details, 100 mL of the 50 mg/L $CoCl_2$ solution was added into the feed side. Subsequently, the salt solutions were filtered through the membranes under a pressure of 1 bar. 30 mL filtrates were collected after 10 min when the filtration process reached steady. Then, removed the residual salt solution, rinsed the AH-rGO membrane with DI water and ammonium hydroxide in the feed side for surface cleaning, after which added another ~50 mL of DI water into the feed side for filtration cleaning. Repeated the filtration process three times.

As shown in Figure S6, after filtration and membrane cleaning three times, the water permeances decreased slightly from 68.6 L m^{-2} h^{-1} bar^{-1} to 58.0 L m^{-2} h^{-1} bar^{-1}, while the corresponding rejection rates were still up to 99.9%. Therefore, the AH-rGO membranes showed outstanding antifouling performance.

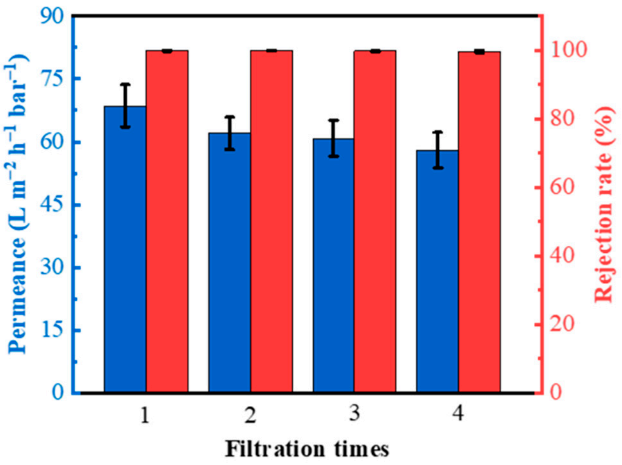

Figure S6. Antifouling performance measurements of the AH-rGO membrane. The permeance and rejection rate of AH-rGO membrane for the 50 mg/L $CoCl_2$ solution after surface and filtration cleaning with DI water.

References

43. Nan, Q.; Li, P.; Cao, B. Fabrication of positively charged nanofiltration membrane via the layer-by-layer assembly of graphene oxide and polyethylenimine for desalination. *Appl. Surf. Sci.* **2016**, *387*, 521–528, https://doi.org/10.1016/j.apsusc.2016.06.150.
44. Zhang, Y.; Zhang, S.; Gao, J.; Chung, N.T.-S. Layer-by-layer construction of graphene oxide (GO) framework composite membranes for highly efficient heavy metal removal. *J. Membr. Sci.* **2016**, *515*, 230–237, https://doi.org/10.1016/j.memsci.2016.05.035.
45. Mahdavi, H.; Razmi, F.; Shahalizade, T. Polyurethane TFC nanofiltration membranes based on interfacial polymerization of poly(bis-MPA) and MDI on the polyethersulfone support. *Sep. Purif. Technol.* **2016**, *162*, 37–44, https://doi.org/10.1016/j.seppur.2016.02.018.
46. Gherasim, C.-V.; Hancková, K.; Palarčík, J.; Mikulášek, P. Investigation of Cobalt(II) Retention from Aqueous Solutions by a Polyamide Nanofiltration Membrane. Journal of Membrane Science 2015, 490, 46–56.
47. Zhang, H.; Zhu, S.; Yang, J.; Ma, A.; Chen, W. Enhanced removal efficiency of heavy metal ions by assembling phytic acid on polyamide nanofiltration membrane. *J. Membr. Sci.* **2021**, *636*, 119591, https://doi.org/10.1016/j.memsci.2021.119591.
48. Moradi, G.; Zinadini, S.; Rajabi, L.; Derakhshan, A.A. Removal of heavy metal ions using a new high performance nanofiltration membrane modified with curcumin boehmite nanoparticles. *Chem. Eng. J.* **2020**, *390*, 124546, https://doi.org/10.1016/j.cej.2020.124546
20. Soyekwo, F.; Zhang, Q.; Gao, R.; Qu, Y.; Lv, R.; Chen, M.; Zhu, A.; Liu, Q. Metal in Situ Surface Functionalization of Polymer-Grafted-Carbon Nanotube Composite Membranes for Fast Efficient Nanofiltration. *J. Mater. Chem. A* **2017**, *5*, 583–592.
31. He, Y.; Miao, J.; Chen, S.; Zhang, R.; Zhang, L.; Tang, H.; Yang, H. Preparation and characterization of a novel positively charged composite hollow fiber nanofiltration membrane based on chitosan lactate. *RSC Adv.* **2019**, *9*, 4361–4369, https://doi.org/10.1039/c8ra09855g.
49. Belkhouche, N.-E.; Merad, N.S.; Mesli, M.; Sefrou, Z. Separation of cobalt and nickel by nanofiltration using a FilmTec membrane. *Euro-Mediterranean J. Environ. Integr.* **2018**, *3*, 12, https://doi.org/10.1007/s41207-018-0051-3.
50. Chen, D.; Zhao, X.; Li, F. Treatment of low level radioactive wastewater by means of NF process. *Nucl. Eng. Des.* **2014**, *278*, 249–254, https://doi.org/10.1016/j.nucengdes.2014.08.001.
41. Zhang, M.; Guan, K.; Ji, Y.; Liu, G.; Jin, W.; Xu, N. Controllable ion transport by surface-charged graphene oxide membrane. *Nat. Commun.* **2019**, *10*, 1–8, https://doi.org/10.1038/s41467-019-09286-8.
51. Gui, L.; Dong, J.; Fang, W.; Zhang, S.; Zhou, K.; Zhu, Y.; Zhang, Y.; Jin, J. Ultrafast Ion Sieving from Honeycomb-like Polyamide Membranes Formed Using Porous Protein Assemblies. *Nano Lett.* **2020**, *20*, 5821–5829, https://doi.org/10.1021/acs.nanolett.0c01350.
52. Yang, H.; Yang, L.; Wang, H.; Xu, Z.; Zhao, Y.; Luo, Y.; Nasir, N.; Song, Y.; Wu, H.; Pan, F.; et al. Covalent organic framework membranes through a mixed-dimensional assembly for molecular separations. *Nat. Commun.* **2019**, *10*, 1–10, https://doi.org/10.1038/s41467-019-10157-5.
27. Dai, F.; Yu, R.; Yi, R.; Lan, J.; Yang, R.; Wang, Z.; Chen, J.; Chen, L. Ultrahigh water permeance of a reduced graphene oxide nanofiltration membrane for multivalent metal ion rejection. *Chem. Commun.* **2020**, *56*, 15068–15071, https://doi.org/10.1039/d0cc06302a.
39. Dai, F.; Zhou, F.; Chen, J.; Liang, S.; Chen, L.; Fang, H. Ultrahigh water permeation with a high multivalent metal ion rejection rate through graphene oxide membranes. *J. Mater. Chem. A* **2021**, *9*, 10672–10677, https://doi.org/10.1039/d1ta00647a.
42. Hu, R.; Zhao, G.; He, Y.; Zhu, H. The Application Feasibility of Graphene Oxide Membranes for Pressure-Driven Desalination in a Dead-End Flow System. *Desalination* **2020**, *477*, 114271.
